# The Relationship between Nutrition Knowledge and Nutrition Facts Table Use in China: A Structural Equation Model

**DOI:** 10.3390/ijerph18126307

**Published:** 2021-06-10

**Authors:** Zeying Huang, Beixun Huang, Jiazhang Huang

**Affiliations:** Institute of Food and Nutrition Development, Ministry of Agriculture and Rural Affairs, Beijing 100081, China; huangzeying@caas.cn (Z.H.); wqtgzy8n899@163.com (B.H.)

**Keywords:** nutrition knowledge, nutrition label, food label, structural equation modeling, China

## Abstract

Since 2013, China has implemented a nutrition label regulation that aims to provide essential nutrition information through nutrition facts tables labeled on the back of food packages. Yet, the relationship between people’s nutrition knowledge and their nutrition label use remains less clear. This study adopted the structural equation modeling approach to analyze a nationally representative survey of 1500 Chinese individuals through the cognitive processing model, interrelated nutrition knowledge, attention to nutrition information on the nutrition facts table, comprehension of nutrition information, food choice and dietary intake. It was found that nutrition knowledge positively influenced attention to nutrition information; a better comprehension of nutrition information, which could benefit healthier food choices, did not relate to a higher level of attention to that information; dietary intake was affected significantly by nutrition knowledge, but it had little impact on food choice. The results signify that nutrition knowledge hardly supports nutrition facts table use among the Chinese people, mainly due to incomprehensible labeled information. Therefore, it emphasizes the need to enhance people’s comprehension through front-of-package labels and corresponding smartphone applications.

## 1. Introduction

A nutrition label, as an effective policy instrument for health promotion and the prevention of non-communicable diseases (NCDs) [1], is expected to help consumers make informed food choices with information on the nutritional values of products [2]. A nutrition facts table is an important nutrition label in China. In 2013, China implemented General Rules of National Prepackaged Food Nutrition Labels (GB 28050-2013) [3] for the mandatory provision of energy value and the amounts of protein, fat, carbohydrates and sodium, as well as the percentages of Nutrient Reference Values (NRV) per 100 g (mL) of food products, on nutrition facts tables.

Nutrition knowledge, broadly defined, refers to knowledge of the concepts and processes related to nutrition and health, including diet and health, diet and disease, dietary guidelines and recommendations [4]. Nutrition knowledge has been reported to be positively associated with diet quality [5], which is regarded as a means of encouraging consumers to make a healthy choice [6]. There is no consensus on the relationship between nutrition knowledge and nutrition label use. To be specific, nutrition knowledge played a positive role in nutrition label use in Greece [7], the United States [8], Switzerland [9], Spain [10], Iran [11] and Ecuador [12]. On the other hand, a few studies found that nutrition label use may lead some consumers to improve their nutrition knowledge with respect to the perceived healthiness of products [13]. However, some have argued that nutrition label use has no significant correlation with nutrition knowledge, as was seen with respondents in New Jersey [14] and rural youth in South Africa [15]. This disparity in results could be attributed to differences in methods (e.g., empirical investigation, systematic review), samples, type of nutrition label, etc. Additionally, the above studies have included sociodemographic, economic and health-related factors that affect nutrition label use, with nutrition knowledge being one of them, yet few have only assessed the relationship between nutrition knowledge and nutrition label use. Therefore, we employed an online survey in China and the model of cognitive processes to establish such linkages. To the best of our knowledge, this is the first study in China, the country with the largest population in the world, which focuses on the relationship between nutrition knowledge and nutrition facts table use. New findings may shed light on future educational campaigns or policies to promote nutrition label use in other countries.

## 2. Conceptual Framework and Hypotheses

### 2.1. Conceptual Framework

**Figure 1:**
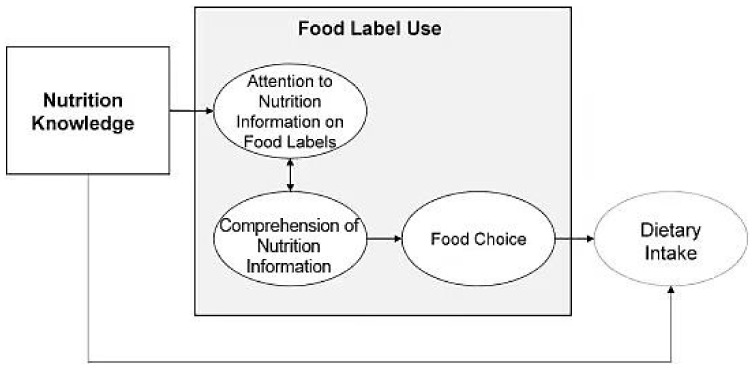
The Cognitive Processing Model. Source: Miller and Cassady [18].

The conceptual model guiding the study is illustrated in Figure 1. Based on the cognitive science literatures (i.e., Feigenbaum [16], Ericsson and Kintsch [17]), Miller and Cassady [18] proposed the cognitive processing model, which is one of the latest theories to present how nutrition knowledge could support food label use on packaged foods. The theory could be applied to three types of food labels: nutrition labels, ingredient lists and health claims [18]. As shown in Figure 1, food label use is expected to influence dietary intake in three aspects: attracting attention to nutrition information, enhancing comprehension of nutrition information and facilitating food choice. Besides influencing dietary intake directly, nutrition knowledge could also impact the previous three aspects, consequently affecting dietary intake indirectly.

Compared with similar theories discussed in Table 1, the cognitive processing model proposes that nutrition label use is not a single behavior, but cognitive processes, which are only impacted by nutrition knowledge. Therefore, the cognitive processing model seems to serve as an appropriate framework to model the relationship between Chinese consumers’ nutrition knowledge and nutrition facts table use.

### 2.2. Hypotheses

According to the cognitive processing model, nutrition knowledge is expected to have a positive effect on the attention to information on nutrition facts tables and dietary intake. Consumers’ comprehension of nutrition information not only interacts with their attention, but also affects food choice, thereby leading to a healthier food intake. Hence, six hypotheses are proposed:

**Hypothesis 1** **(H1).** *Consumers with a high level of nutrition knowledge are more likely to pay attention to information on a nutrition facts table.*

**Hypothesis 2** **(H2).** *Consumers who pay attention to information on a nutrition facts table are more likely to comprehend that information.*

**Hypothesis 3** **(H3).** *Consumers with a better comprehension of information on a nutrition facts table are more likely to pay attention to that information.*

**Hypothesis 4** **(H4).** *Consumers with a better comprehension of information on a nutrition facts table are more likely to make healthier food choices.*

**Hypothesis 5** **(H5).** *Consumers who take into consideration information on a nutrition facts table when purchasing food are more likely to have a healthy dietary intake than those not considering such information.*

**Hypothesis 6** **(H6).** *Consumers with a high level of nutrition knowledge are more likely to have a healthy dietary intake.*

## 3. Methods and Materials

### 3.1. Measures and Collection of Data

With respect to the cognitive processing model, nutrition knowledge, cognitive processes and dietary intake could be measured by a self-administered questionnaire [18]. To assess the five latent variables shown in Table 2, scale items related to nutrition knowledge and dietary intake were obtained from Dietary Guidelines for Chinese Residents, which is an official dietary guideline in China for a balanced diet [22]. The scale items measuring the cognitive processes (i.e., attention to nutrition information on food labels, comprehension of nutrition information and food choice) were based on prior studies [22,23,24,25,26,27]. All items were evaluated on a 5-point Likert scale, ranging from 1 ‘strongly disagree’ to 5 ‘strongly agree’.

This study developed a self-administered questionnaire containing 25 questions including socio-demographic information (see Supplementary Materials for details). From 29 July to 21 August 2020, Chinese participants were recruited to complete an online survey by Wenjuanxing (https://www.wjx.cn/ (accessed on 29 July 2020)), one of the leading companies specializing in online questionnaire and data collection in China. The online platform of Wenjuanxing is widely used by Chinese scholars due to its high-quality online data services. The study employed a proportionate stratified sampling approach to select 50 individuals who were proportionally distributed across age groups according to the age distribution among China’s population—20% for each of the five age groups: below 18 years old, between 18 and 25 years old, between 26 and 30 years old, between 31 and 40 years old and above 40 years old—from each of China’s 30 provinces/autonomous regions (except Tibet). To obtain accuracy, every aspect of the questionnaire was explained in detail to the respondents. This generated 1500 valid samples (i.e., 50 samples × 30 regions) used for analysis.

### 3.2. Methods

Propositions that link the exogenous variables to the endogenous variables were analyzed using structural equation modeling (SEM) which is reliable in examining the relationships between different constructs (i.e., differences among groups on latent variables), and provides accurate and meaningful outcomes [27]. Compared with other techniques, it allows us to create several indicator variables (i.e., observable variables) per construct, which does not require the split analysis method and yields valid and clear inferences [28]. Therefore, results of the relationships among variables are reliable and neutral [29]. In addition, it has the capability to scrutinize complicated associations and a variety of hypotheses by instantly incorporating mean structures and group estimation [30]. Hence, the hypotheses proposed above were analyzed by the structural equation model (SEM). Specifically, data analysis was performed in two stages. In the first stage, reliability analysis was conducted using SPSS (ver. 25.0) to evaluate the stability and consistency of measured items. In the second stage, the evaluation of goodness-of-fit indices for the proposed structural equation model and testifying hypotheses were performed using analysis of moment structure (AMOS, ver. 21.0).

## 4. Results

Our sample is representative of the Chinese population in terms of socio-demographic characteristics. Table 3 summarizes the demographic features of the sampled 1500 individuals. Overall, respondents were predominantly male (58.07%), aged between 18 and 44 years (66.80%), with a junior high school degree (42.40%) and had a middle-income between 10,000 and 50,000 Yuan after tax (21.27%).

### 4.1. Discriminant Validity Analysis

As shown in Table 4, the interrelationships among constructs were checked using Pearson’s correlation test, which generated statistically significant correlations. The discriminant validity issue was examined by the square root of the average variance extracted (AVE). The aim of performing discriminant validity is to evaluate the extent to which the items are not theoretically correlated. The data did not have any discriminant validity issues, as the value of the square root of AVE is higher than its correlation with other constructs [31].

### 4.2. Testing the Fit of the Model

Exploratory factor analysis (EFA) was conducted to examine the reliability and validity of the measurement model. The suitability of data was calculated via the Kaiser–Meyer–Olkin (KMO) test and Bartlett’s test of sphericity (BTS). The condition of EFA was met, as the value of BTS was 826.13. The KMO value was 0.872 (*p* < 0.001), indicating that the sample is suitable for factor analysis as recommended by Kaiser [32]. Composite reliability (CR) test was performed to examine the consistency of all constructs’ items. To evaluate the level to which the items were theoretically associated with each other, the convergent validity test was performed by utilizing AVE and item loadings [33]. Results revealed that AVE values surpassed 0.50 for all constructs, suggesting that the latent constructs retained a minimum of 50% of the variance.

The reliability of the samples was examined by a reliability analysis test. Nunnally [34] advised that the reliability coefficient must not be less than 0.70. Results from this study indicated that the values of CR and Cronbach’s α exceeded 0.70 for all constructs (Table 5) and suggested validity and reliability of the data.

### 4.3. Valuation of Structural Equation and Hypothesis Testing

**Figure 2:**
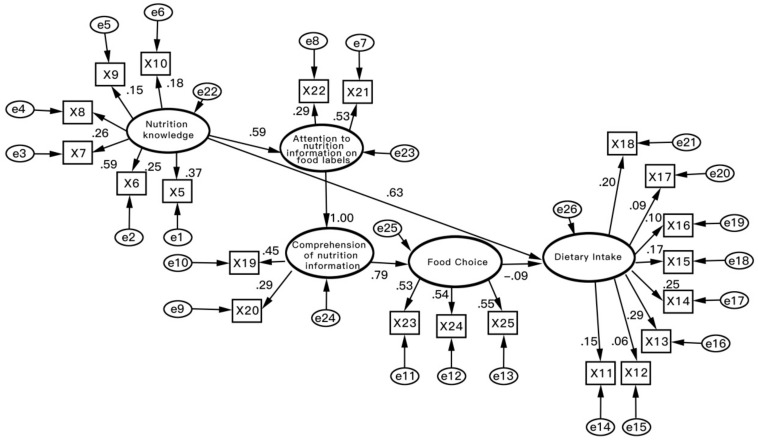
Results of Structural Equation Modeling. Notes: Comparative Fit Index = 0.932; Goodness-of-fit Index = 0.931; Root Mean Square Error of Approximation = 0.059; Degrees of freedom = 184; chi-square = 203.20; x5~x25 is the scale items code and e1~e26 is statistical error of 5 variables and 21 scale items.

The structural model was estimated, and the hypothesized relationships were analyzed after the validity and reliability of the measures were attained. Figure 2 shows the path analysis of the structural model. The goodness-of-fit indices for the structural model were calculated (see Table 6). Each of the fitting index values (SCS = 2.840, CFI = 0.932, IFI = 0.936, GFI = 0.931, AGFI = 0.918, RMSEA = 0.059, NNFI = 0.980, NFI = 0.995, and AIC = 1.867) out-performed the respective threshold value, signifying that the model satisfactorily incorporated the data [31,35,36].

Table 7 shows that Hypothesis 1 was accepted because the path coefficient of H1 (0.59, *p* < 0.001) indicated that consumers’ nutrition knowledge level had a positive and significant effect on their attention to information on a nutrition facts table. The path coefficients failed to validate Hypothesis 2 (1.00, *p* > 0.01) and Hypothesis 3 (0.00, *p* > 0.01), which suggested that consumers’ attention to nutrition information was not related to a good comprehension of nutrition information. Hypothesis 4 (0.79, *p* < 0.001) was accepted and suggested that consumers’ food choice was significantly positively affected by their comprehension of nutrition information. Hypothesis 5 (0.09, *p* > 0.01) was rejected, which suggested that consumers’ food choice through information on a nutrition facts table did not have a significant effect on dietary intake. Hypothesis 6 (0.63, *p* < 0.001) was accepted, indicating that nutrition knowledge played a significant role in consumers’ dietary intake.

## 5. Discussion

In the present study, the behavior of 1500 Chinese population samples’ nutrition facts table use was innovatively subdivided into attention, comprehension and decision making, for identifying the influence path of Chinese nutrition knowledge on nutrition label use, which might contribute to addressing the issues constraining nutrition label use. However, there are some clear limitations to our study which will be solved in future research. For example, the cognitive processing model only focuses on the effect of nutrition knowledge on nutrition label use but lacks the discussion on the reverse effect; moreover, it is a challenge to develop scale items for measuring all variables which the above theoretical model does not specify. We should note that the nutrition knowledge questions seemed far-fetched and could easily be mistaken for measuring respondents’ nutritional awareness, thus more targeted nutrition knowledge questions need to be redesigned. Additionally, scale items measuring the comprehension of and attention to nutrition information on food labels are biased towards subjectivity despite efforts to review literatures; moreover, what we know about individual cognition comes from self-report measures. However, self-report questionnaires are vulnerable to social desirability bias due to respondents’ tendencies to answer in a more socially acceptable way [37]. For accurate research results, more attempts are needed to make thorough complex indirect questioning methods, such as the list experiment.

### 5.1. Association between Nutrition Knowledge and Attention to Information on Nutrition Facts Table

Empirical results showed that respondents with a high level of nutrition knowledge are likely to focus on information on the nutrition facts table, which was in line with the findings of Jones and Richardson [38] and Steinhauser et al. [39] that nutrition knowledge may enable consumers to pay attention to information on the nutrition label. Nutrition facts tables have been implemented for eight years in China yet are still not easily found by most consumers due to the location on the back of packaging. However, those individuals with a high knowledge of nutrition might reflect a basic interest in healthy eating [9] and generally care more about the impact of food intake on their own health, meaning that they tend to be sensitive to specific nutrients’ amounts on the nutrition facts table, which leads them to search that information initiatively.

### 5.2. Association between Attention to and Comprehension of Information on Nutrition Facts Table

As Miller and Cassady [18] reported, attention to nutrition information on food labels may be associated with understanding of that information. Nevertheless, our study did not show such a relation. One possible reason could be that information on Chinese nutrition facts tables is complex and incomprehensible, especially the concept and function of NRV. Thus, people who know little about such information might find it difficult to make decisions based on information on that label. Additionally, nutrition facts tables are common in many other countries [40]. By contrast, less nutrients (i.e., carbohydrate, protein, fat and sodium) mandatorily provided on the nutrition facts tables in China [41] scarcely meet the demand of all consumers who are possibly not concerned about that information even if they already know enough about it.

### 5.3. Association between Comprehension of Nutrition Information and Food Choice

Our data confirmed that consumers who understand nutrition facts table information well could make healthy food choices. It proves that information communicated to consumers by nutrition facts tables in China is useful, which could provide strong support for switching to high-protein foods, low-fat foods and low-sodium foods on the condition that consumers gain a good understanding of that information. However, some evidence suggests that a nutrition facts table as a non-interpretative label is less likely to help consumers make quick purchase decisions [42]. In particular, almost all consumers rely on their shopping habits at the time of purchase if they are not well informed of the information on nutrition facts tables, which may eventually make the nutrition label superfluous. Therefore, consumers’ comprehension of information on the nutrition facts table should be paid great attention to, because it is a crucial cognitive aspect linking attention with food choice. Otherwise, it could be a barrier to nutrition label use. This conclusion is accordance with the studies of Besler et al. [43] and Gorton et al. [44]. They insisted that a lack of understanding of nutrition facts on food labels is a key reason for consumers not to use nutrition labels.

### 5.4. Association between Food Choice and Dietary Intake

Unexpectedly, consumers’ food-related decisions through information on a nutrition facts table did not yield a significant effect on dietary intake. This could be partially due to different goals between the nutrition facts tables and The Chinese Dietary Guidelines. To be specific, the aim of the nutrition facts table in China is to improve residents’comprehension level of an appropriate daily intake of carbohydrate, protein, fat and sodium from a specific product, while The Chinese Dietary Guidelines as an official dietary intake regulation is used to advocate a balanced diet and rational food choice. Another possible reason is that current nutrition facts table generate limited guidance of residents’ dietary intake, given that it is only applied to prepackaged food which is a part of food source in China [45].

### 5.5. Association between Nutrition Knowledge and Dietary Intake

The results showed a significant effect of nutrition knowledge on dietary intake, which was in accordance with previous studies of Ahmadi et al. [11] and Breen et al. [46], that dietary intake is directly impacted by nutrition knowledge. In China, nutrition knowledge, which is commonly acquired from The Chinese Dietary Guidelines, recommends a balanced diet and is often regarded as an important instrument for dietary intake. However, the relationship between nutrition knowledge and nutrition facts table use is found to be insignificant because consumers’ high level of nutrition knowledge only aroused attention to the information on the nutrition facts table, but failed to facilitate nutrition label use due to an insignificant impact on the comprehension of that information. This highlights the importance of increasing Chinese residents’ understanding of information on nutrition facts tables.

## 6. Conclusions and Recommendations

Through online survey data collected from 1500 people across China, this study provided deeper insights for examining the relationship between nutrition knowledge and nutrition facts table use with the cognitive processing model. The results suggested that nutrition knowledge could not support consumers’ use of nutrition facts table in China due to no associations between them. Nutrition knowledge was found to positively influence attention to that information and to dietary intake, but no mutual influence existed between attention to and comprehension of that information. Healthy products were purchased by consumers who had a good understanding of the information on nutrition facts tables, whereas dietary intake was hardly affected by food decision making.

The following policy recommendations are offered: (1) national targeted education campaigns regarding the interpretation of nutrition facts tables need to be implemented in order to increase residents’ comprehension of the concept, function and application method of each nutrient and NRV%; (2) development of front-of-package (FOP) labels need to be considered. FOP labels that use graphics and colors to depict food nutrient contents have increasingly become an important policy option [47]. Practice has shown that information on FOP labels is easier to understand than that on nutrition facts table [48,49]. Recently, the United States, the United Kingdom, France, Australia and other countries have implemented FOP labels (e.g., multiple traffic light signpost labels, and health star rating system), but China hasn’t yet carried out FOP labels. Therefore, it is necessary to improve the nutrition label format and design a FOP label suitable for China; (3) smartphone applications of nutrition facts tables should be promoted. There is evidence that smartphone applications as a convenient information technology, with a barcode scanning function, could be designed to interpretate nutrition facts table information [50,51]. We call for the development of relevant smartphone applications to assist consumers in food selection.

## Figures and Tables

**Table 1:** Overview of the theories conceptualizing the relationship between nutrition knowledge and nutrition label use.

References	The Theory	The Relationship between Nutrition Knowledge and Nutrition Label Use
Grunert and Wills [19]	Consumer behavior and food choice theory	Nutrition knowledge could impact nutrition label use but indirectly by the means of many factors (i.e., search, exposure, perception, liking and understanding of nutrition information on food label).
Drichoutis et al. [20]	External consumer information	Nutrition label use could be influenced by a large number of factors including nutrition knowledge.
Hess et al. [9]	The comprehensive model of determinants of label use	Nutrition knowledge as a primary motivator could have effect on frequency of nutrition label use.
Rimpeekool et al. [21]	Knowledge-Attitude-Behaviour and Health Belief mixed model (KAB-HBM)	Nutrition knowledge could influence nutrition label use but indirectly by attitude (diet-health awareness).
Miller and Cassady [18]	The cognitive processing model	Nutrition knowledge as only one determinant could directly influence nutrition label use which contains cognitive processes.

**Table 2:** Variables, scale items and references.

Latent Variables		Scale Items	Mean	References	
Nutrition knowledge		I know that the diet should be varied and grain-based.	2.31	Chinese Nutrition Society [22]	
		I know how to have a balanced diet and maintain a healthy weight.	3.09		
		I know how to have more fruits, vegetables, dairy and soy.	1.80		
		I know how to have fish, poultry, eggs and lean meat in moderation.	2.04		
		I know how to have less salt, oil, sugar and alcohol.	2.88		
		I know how to eliminate waste and try new things.	2.10		
Dietary intake		I ate 12 kinds of food today.	1.88	Chinese Nutrition Society [22]	
		I eat staple food at every meal.	2.45		
		I ate more than 4 kinds of fruits and vegetables today.	3.02		
		I had at least 1 serving of milk or yogurt today.	2.99		
		I ate at least 1 serving of beans or soy products today.	3.50		
		I ate more than 5 servings of fish this week (about 40–50 g of edible portion per serving).	2.75		
		I ate 5–10 servings of poultry and livestock this week (about 40–50 g per serving).	3.02		
		I ate 4–7 eggs this week.	2.88		
Attention to information on the nutrition facts table		I pay attention to the nutrients on the nutrition facts table when shopping.	3.26	Cannoosamy et al., [23]	
		I pay attention to the nutrient contents on the nutrition facts table when shopping.	2.78		
Comprehension of information on nutrition facts table		I know the contents on the nutrition facts table.	2.55	Swartz et al., [24] Haasova and Florack [25]	
		I know the format of nutrition facts table.	3.08		
Food choice		I buy high-protein foods every week or month ^a^	2.88	Volkova and Mhurchu [26] Graham and Roberto [27]	
		I buy low-fat foods every week or month ^b^	3.02		
		I buy low-sodium foods every week or month ^c^	1.92		

Note: ^a^ High-protein foods are those containing more than 12 g protein per 100 g or 6 g per 100 mL, such as high-protein chicken breast and high-protein milk. ^b^ Low-fat foods are those whose fat is less than 3 g per 100 g or 1.5 g per 100 mL, such as low-fat beef jerky and low-fat milk. ^c^ Low-sodium foods are those with less than 5% of the recommended daily intake of nutrients for sodium, such as low-sodium edible salt and low-sodium soy sauce.

**Table 3:** Demographic characteristics of the sample.

Characteristics	Classifications	N	Percentage%
Gender	Male	629	58.07
	Female	871	41.93
Age	<18	300	20.00
	from 18 to 44	1002	66.80
	from 45 to 59	189	12.60
	≥60	9	0.60
Education level	Primary school and below	3	0.20
	Junior high school	36	42.40
	High school	373	34.87
	College/Bachelor	992	18.13
	Postgraduate or above	96	4.40
Annual household income (after tax)	<10,000 Yuan	127	8.47
	From 10,000 Yuan to 50,000 Yuan	319	21.27
	From 50,000 Yuan to 100,000 Yuan	315	21.00
	From 100,000 Yuan to 150,000 Yuan	299	19.92
	From 150,000 Yuan to 200,000 Yuan	226	15.07
	≥200,000 Yuan	214	14.27

Note: One US dollar is equal to 6.941 Chinese Yuan and One Euro is equal to 8.199 Chinese Yuan from 29 July to 21 August 2020.

**Table 4:** Factor correlations and discriminant validity.

Factors	Nutrition Knowledge	Dietary Intake	Comprehension of Information on Nutrition Facts Table	Attention to Information on Nutrition Facts Table	Food Choice
Nutrition knowledge	[0.711]				
Dietary intake	0.839 ***	[0.798]			
Comprehension of information on nutrition facts table	0.604 *	0.620 *	[0.740]		
Attention to information on nutrition facts table	0.533 ***	0.543 *	0.722 *	[0.730]	
Food choice	0.597 *	0.782 *	0.731 ***	0.620 *	[0.772]

Notes: Values in brackets [] indicate the square root of AVEs. A significance level (*** *p* < 0.001, * *p* < 0.05).

**Table 5:** Factor loadings and convergent validity results.

Variables	Scale Items Code	Scale Items	Standard Loadings	AVE	Composite Reliability	Cronbach’ s α
Nutrition knowledge	X5	I know that the diet should be varied and grain-based.	0.520	0.506	0.801	0.799
	X6	I know to have a balanced diet and maintain a healthy weight.	0.518			
	X7	I know to have more fruits, vegetables, dairy and soy.	0.808			
	X8	I know to have fish, poultry, eggs and lean meat in moderation.	0.707			
	X9	I know to have less salt, oil, sugar and alcohol.	0.592			
	X10	I know to put an end to waste and promote new food fashion.	0.587			
Dietary intake	X11	I ate 12 kinds of food today.	0.591	0.637	0.830	0.738
	X12	I eat staple food at every meal.	0.523			
	X13	I ate more than 4 kinds of fruits and vegetables today.	0.674			
	X14	I had at least 1 serving of milk or yogurt today.	0.575			
	X15	I ate at least 1 serving of beans or soy products today.	0.550			
	X16	I ate more than 5 servings of fish this week (about 40–50 g of edible portion per serving).	0.574			
	X17	I ate 5–10 servings of poultry and livestock this week (about 40–50 g per serving).	0.681			
	X18	I ate 4–7 eggs this week.	0.501			
Comprehension of nutrition information	X19	I know the contents on the nutrition facts table.	0.593	0.548	0.805	0.771
	X20	I know the format of the nutrition facts table.	0.550			
Attention to nutrition information on food labels	X21	I pay attention to the nutrients on the nutrition facts table when shopping.	0.579	0.533	0.752	0.705
	X22	I pay attention to the nutrient content on the nutrition facts table when shopping.	0.575			
Food choice	X23	I buy high-protein foods every week or every month.	0.611	0.596	0.702	0.759
	X24	I buy low-fat foods every week or every month.	0.632			
	X25	I buy low-sodium foods every week or every month.	0.629			

Notes: Rotation technique: Promax; extraction technique: maximum likelihood; total variance elucidated: 62.51%; Bartlett’s test of sphericity: χ2 = 826.13; Kaiser–Meyer–Olkin measure of sampling adequacy: 0.872 (*p* < 0.001).

**Table 6:** Structural equation modeling fitting.

Goodness-of-Fit Indices	Fitting Index Values	Fitting
Standard Chi—Square (SCS)	2.840	<3, good
Comparative Fit Index (CFI)	0.932	>0.9, good
Incremental Fit Index (IFI)	0.936	>0.9, good
Goodness-of-fit Index (GFI)	0.931	>0.9, good
Adjusted Goodness-of-fit Index (AGFI)	0.918	>0.9, good
Root Mean Square Error of Approximation (RMSEA)	0.059	<0.08, good
Non-Normalizing Fitting Index (NNFI)	0.980	>0.9, good
Norm Fitting Index (NFI)	0.995	>0.9, good
Akek Information Standard (AIC)	1.867	<2, good

**Table 7:** Test results of the hypothesis.

Hypothesized Paths	Normalized Path Coefficient	*T* Value	Accepted
H1: Nutrition knowledge→Attention to nutrition information on nutrition facts table	0.59 ***	8.993	Yes
		
H2: Attention to nutrition information on nutrition facts table→Comprehension of nutrition information	1.00	0.910	No
		
H3: Comprehension of nutrition information→Attention to nutrition information on nutrition facts table	0.00	0.000	No
		
H4: Comprehension of nutrition information→Food choice	0.79 ***	6.984	Yes
		
H5: Food choice→Dietary intake	0.09	1.010	No
H6: Nutrition knowledge→Dietary intake	0.63 ***	5.981	Yes

Notes: a significance level (*** *p* < 0.001, * *p* < 0.05).

## Supplementary Materials

The following are available online at https://www.mdpi.com/article/10.3390/ijerph18126307/s1: the questionnaire.

## References

[1] World Health Organization (2004). Global Strategy on Diet, Physical Activity and Health.

[2] Campos S., Doxey J., Hammond D. (2011). Nutrition labels on pre-packaged foods: A systematic review. Public Health Nutr..

[3] Ministry of Health (MoH), PRC (2011). General Rules for Nutrition Labeling of Pre-Packaged Food.

[4] Loretta M.K., Katrina G., Gavin T. (2014). The contribution of three components of nutrition knowledge to socio-economic differences in food purchasing choices. Public Health Nutr..

[5] Geaney F., Fitzgerald S., Harrington J.M., Kelly C., Greiner B.A., Perry I.J. (2015). Nutrition knowledge, diet quality and hypertension in a working population. Prev. Med. Rep..

[6] Deshmukh N., Goyal R. (2017). Food label reading knowledge and understanding among consumers. Int. J. Nutr. Pharmacol. Neurol. Dis..

[7] Dichoutis A.C., Lazaridis P., Nayga R.M. (2005). Nutrition knowledge and consumer use of nutritional food labels. Eur. Rev. Agric. Econ..

[8] Fitzgerald N., Damio G., Segura-Pérez S., Pérez-escamilla R. (2008). Nutrition knowledge, food label use, and food intake patterns among Latinas with and without type 2 diabetes. J. Am. Diet. Assoc..

[9] Hess R., Visschers V.H.M., Siegrist M. (2011). The role of health-related, motivational and socio-demographic aspects in predicting food label use: A comprehensive study. Public Health Nutr..

[10] Carrillo E., Varela P., Fiszman S. (2012). Influence of nutritional knowledge on the use and interpretation of Spanish nutritional food labels. J. Food Sci..

[11] Ahmadi A., Torkamani P., Sohrabi Z., Ghahremani F. (2013). Nutrition knowledge: Application and perception of food labels among women. Pak. J. Biol. Sci..

[12] Orozco F., Ochoa D., Muquinche M., Padro M., Melby C.L. (2017). Awareness, comprehension, and use of newly-mandated nutrition labels among Mestiza and Indigenous Ecuadorian women in the central andes region of Ecuador. Food Nutr. Bull..

[13] Oostenbach L.H., Slits E., Robinson E., Sacks G. (2019). Systematic review of the impact of nutrition claims related to fat, sugar and energy content on food choices and energy intake. BMC Public Health.

[14] Nayga R.M., Lipinski D., Savur N. (1998). Consumers’ use of nutritional labels while food shopping and at home. J. Consum. Aff..

[15] Xazela N., Chinyamurindi W.T., Shava H. (2019). The relationship between nutrition reading and label use and nutrition knowledge amongst a sample of rural youth studying at a university in South Africa. Health SA Gesondheid.

[16] Feigenbaum E.A., Klahr D., Kotovsky K. (1989). What hath simon wrought?. Complex Information Processing; The Impact of Herbert A. Simon.

[17] Ericsson K.A., Kintsch W. (1995). Long-term working memory. Psychol. Rev..

[18] Miller L.M.S., Cassady D.L. (2015). The effects of nutrition knowledge on food label use. A review of the literature. Appetite.

[19] Grunert K.G., Wills J. (2007). A review of European research on consumer response to nutrition information on food labels. J. Public Health.

[20] Drichoutis A.C., Lazaridis P., Nayga R.M., Kapsokefalou M., Chryssochoidis G. (2008). A theoretical and empirical investigation of nutritional label use. Eur. J. Health Econ..

[21] Rimpeekool W., Banwell C., Seubsman S.-A., Kirk M.D., Yieng-Sbirakos V., Sleigh A.C. (2016). “I rarely read the label”: Factors that influence Thai consumer responses to nutrition labels. Glob. J. Health Sci..

[22] Chinese Nutrition Society (2016). Dietary Guidelines for Chinese Residents.

[23] Cannoosamy K., Pugo-Gunsam P., Jeewon R. (2014). Consumer knowledge and attitudes toward nutritional labels. J. Nutr. Educ. Behav..

[24] Swartz J.J., Dowray S., Braxton D., Mihas P., Viera A.J. (2013). Simplifying healthful choices: A qualitative study of a physical activity based nutrition label format. J. Nutr..

[25] Haasova S., Florack A. (2019). Sugar labeling: How numerical information of sugar content influences healthiness and tastiness expectations. PLoS ONE.

[26] Volkova E., Mhurchu C.N. (2015). The influence of nutrition labeling and point-of-purchase information on food behaviours. Cur. Obes. Rep..

[27] Graham D.J., Roberto C.A. (2016). Evaluating the impact of U.S. food and drug administration–proposed nutrition facts label changes on young adults’visual attention and purchase intentions. Health Educ. Behav..

[28] Lei P., Wu Q. (2007). Introduction to structural equation modeling: Issues and practical considerations. Educ. Meas. Issues Pract..

[29] Neale M.C., Hunter M.D., Pritikin J.N., Zahery M., Brick T.R., Kirkpatrick R.M., Estabrook R., Bates T.C., Maes H.H., Boker S.M. (2016). OpenMx20: Extended structural equation and statistical modeling. Psychometrika.

[30] Al-Gahtani S.S. (2016). Empirical investigation of e-learning acceptance and assimilation: A structural equation model. Appl. Comput. Inf..

[31] Kline R.B. (2005). Principles and Practice of Structural Equation Modeling.

[32] Kaiser H.F. (1974). An index of factorial simplicity. Psychometrika.

[33] Anderson J.C., Gerbing D.W. (1988). Structural equation modeling in practice: A review and recommended two-step approach. Psychol. Bull..

[34] Nunnally J.C. (1978). Psychometric Theory.

[35] Thompson B. (2004). Exploratory and Confirmatory Factor Analysis: Understanding Concepts and Applications.

[36] Byrne B.M. (1994). Structural Equation Modeling with EQS and EQS/Windows: Basic Concepts, Applications, and Programming.

[37] Kim S.H., Kim S. (2016). National culture and social desirability bias in measuring public service motivation. Admin. Soc..

[38] Jones G., Richardson M. (2007). An objective examination of consumer perception of nutrition information based on healthiness ratings and eye movements. Public Health Nutr..

[39] Steinhauser J., Janssen M., Hamm U. (2019). Who buys products with nutrition and health claims? a purchase simulation with eye tracking on the influence of consumers’ nutrition knowledge and health motivation. Nutrients.

[40] Miller L.M.S., Cassady D.L. (2012). Making healthy food choices using nutrition facts panels. The roles of knowledge, motivation, dietary modifications goals, and age. Appetite.

[41] Peng Z., Li Y., Yan L. (2018). The impact of the nutrition labels regulation on firm marketing action and performance in China. J. Public Policy Mark..

[42] Liu R., Hoefkens C., Verbeke W. (2015). Chinese consumers’ understanding and use of a food nutrition label and their determinants. Food Qual. Prefer..

[43] Besler H.T., Buyuktuncer Z., Uyar M.F. (2012). Consumer understanding and use of food and nutrition labelling in Turkey. J. Nutr. Educ. Behav..

[44] Gorton D., Mhurchu C.N., Chen M.H., Dixon R. (2009). Nutrition labels: A survey of use, understanding and preferences among ethnically diverse shoppers in New Zealand. Public Health Nutr..

[45] He Y., Huang L., Yan S., Li Y., Lu L., Wang H., Niu W., Zhang P. (2018). Awareness, understanding and use of sodium information labelled on pre-packaged food in Beijing: A cross-sectional study. BMC Public Health.

[46] Breen C., Ryan M., Gibney M.J., O’Shea D. (2015). Diabetes-related nutrition knowledge and dietary intake among adults with type 2 diabetes. Br. J. Nutr..

[47] Neal B., Crino M., Dunford E., Gao A., Greenland R., Li N., Ngai J., Mhurchu C., Pettigrew S., Sacks G. (2017). Effects of different types of front-of-pack labelling information on the healthiness of food purchases—a randomised controlled trial. Nutrients.

[48] Bix L., Sundar R.P., Bello N.M., Peltier C., Weatherspoon L.J., Becker M.W. (2015). To see or not to see: Do front of pack nutrition labels affect attention to overall nutrition information?. PLoS ONE.

[49] Fatmah R.D. (2019). The impact of front-of-package traffic light (FoPTL) in the senior high school students’ nutrition labels comprehension. Curr. Res. Nutr. Food Sci. J..

[50] Kulyukin V.A., Zaman T., Andhavarapu S.K. Effective Nutrition Label Use on Smartphones. Proceedings of the 2014 International Conference on Internet Computing and Big Data.

[51] Zaman T. (2016). Vision Based Extraction of Nutrition Information from Skewed Nutrition Labels.

