# Supplementary material for: The Relationship between Nutrition Knowledge and Nutrition Facts Table Use in China: A Structural Equation Model
Source: Int J Environ Res Public Health. 2021 Jun 10;18(12):6307. doi: 10.3390/ijerph18126307 (PMC8296123; doi:10.3390/ijerph18126307)
Supplement: Supplementary file 1 [file ijerph-18-06307-s001.zip › ijerph-1217922-supplementary.pdf]

Supplementary Materials : The questionnaire

Section 1 Nutrition knowledge

Q1-1 I know that the diet should be varied and grain-based.

A.strongly disagree B. disagree C.neutral D.agree E.strongly agree

Q1-2 I know to have a balanced diet and maintain a healthy weight.

A.strongly disagree B. disagree C.neutral D.agree E.strongly agree

Section 2 Dietary intake

Q2-1 I know to have more fruits, vegetables, dairy and soy.

A.strongly disagree B. disagree C.neutral D.agree E.strongly agree

Q2-2 I know to have fish, poultry, eggs and lean meat in moderation.

A.strongly disagree B. disagree C.neutral D.agree E.strongly agree

Q2-3 I know to have less salt, oil, sugar and alcohol.

A.strongly disagree B. disagree C.neutral D.agree E.strongly agree

Q2-4 I know how to eliminate waste and try new things.

A.strongly disagree B. disagree C.neutral D.agree E.strongly agree

Q2-5 I ate 12 kinds of food today.

A.strongly disagree B. disagree C.neutral D.agree E.strongly agree

Q2-6 I eat staple food at every meal.

A.strongly disagree B. disagree C.neutral D.agree E.strongly agree

Q2-7 I ate more than four kinds of fruits and vegetables today.

A.strongly disagree B. disagree C.neutral D.agree E.strongly agree

Q2-8 I had at least 1 serving of milk or yogurt today.

A.strongly disagree B. disagree C.neutral D.agree E.strongly agree

Q2-9 I ate at least 1 serving of beans or soy products today.

A.strongly disagree B. disagree C.neutral D.agree E.strongly agree

Q2-10 I ate more than 5 servings of fish this week. (about 40 ~ 50g of edible portion per serving)

A.strongly disagree B. disagree C.neutral D.agree E.strongly agree

Q2-11 I ate 5~ 10 servings of poultry and livestock this week. (about 40~ 50g per serving)

A.strongly disagree B. disagree C.neutral D.agree E.strongly agree

Q2-12 I ate 4~ 7 eggs this week.

A.strongly disagree B. disagree C.neutral D.agree E.strongly agree

Section 3 Comprehension of nutrition information

Q3-1 I know contents on nutrition label.

A.strongly disagree B. disagree C.neutral D.agree E.strongly agree

Q3-2 I know the format of nutrition label.

A.strongly disagree B. disagree C.neutral D.agree E.strongly agree

Section 4 Attention to nutrition information on food labels

Q4-1 I pay attention to the nutrients on the nutrition label when shopping.

A.strongly disagree B. disagree C.neutral D.agree E.strongly agree

Q4-2 I pay attention to the nutrient contents on nutrition label when shopping.

A.strongly disagree B. disagree C.neutral D.agree E.strongly agree

Section 5 Food choice

Q5-1 I buy high-protein foods every week or month. (High-protein foods are those foods whose protein are more than 12g per 100g or 6g per 100mL, such as high protein chicken breast and high protein milk.)

A.strongly disagree B. disagree C.neutral D.agree E.strongly agree

Q5-2 I buy low-fat foods every week or month.(Low-fat foods are those foods whose protein are less than 3g per 100g or 1.5g per 100mL, such as low-fat beef jerky and low fat milk.)

A.strongly disagree B. disagree C.neutral D.agree E.strongly agree

Q5-3 I buy low-sodium foods every week or month.(Low-sodium foods are those foods with percentage of the recommended daily intake of nutrients for sodium less than 5%, such as low-sodium edible salt and low-sodium soy sauce.)

A.strongly disagree B. disagree C.neutral D.agree E.strongly agree

Section 6 Demographic characteristics

Q6-1 Your gender : A. Male B. Female

Q6-2 Your age: A. under 18 years old B. 18-44 years old C.45-59 years old D. 60 years old and above

Q6-3 Your education level: A. Primary school or below B. Junior school C. Senior school D. Junior college or undergraduate E. Postgraduate or above

Q6-4 Your individual annual income on average (Yuan):

A.≤10,000 B.10,001-50,000 C.50,001-100,000 D.100,001-150,000 E.150,001-200,000 F.≥200,001
